# Signatures of exposure to childhood trauma in young adults in the structure and neurochemistry of the superior temporal gyrus

**DOI:** 10.1177/02698811231168243

**Published:** 2023-04-17

**Authors:** Piril Hepsomali, Sandra Machon, Holly Barker, David J Lythgoe, Kenneth Hugdahl, Maria Gudbrandsen, Paul Allen

**Affiliations:** 1 Department of Psychology, Roehampton University, London, UK; 2 Department of Psychology, Royal Holloway, Combined Universities Brain Imaging Centre, University of London, Surrey, UK; 3 Department of Neuroimaging, Centre for Neuroimaging Sciences, King’s College London, Institute of Psychiatry, Psychology & Neuroscience, London, UK; 4 Department of Biological and Medical Psychology, University of Bergen, Bergen, Norway; 5 Department of Psychosis Studies, King’s College London, Institute of Psychiatry, Psychology and Neuroscience, London, UK

**Keywords:** GABA, glutamate, spectroscopy, childhood trauma, grey matter, early life adversity

## Abstract

**Background:**

Childhood trauma (CT) has been linked to increased risk for mental illness in adulthood. Although work in experimental animals has shown that early life stressors can affect inhibitory and excitatory neurotransmission in adult rodents, with possible excitotoxic effects on local grey matter volumes (GMV), the neurobiological mechanisms that mediate this relationship in humans remain poorly understood.

**Aim:**

To examine glutamate and gamma-aminobutyric acid (GABA) metabolite concentrations and potential excitotoxic effects on GMV, in adults who experienced CT.

**Methods:**

Fifty-six young adults (*M*_age_ = 20.41) were assigned to High CT (*n* = 29) and Low CT (*n* = 27) groups (by using the CT questionnaire) and underwent magnetic resonance spectroscopy (^1^H-MRS) to measure temporal lobe metabolite concentrations and volumetric imaging to measure GMV.

**Results:**

Glutamate concentrations did not differ between groups; however, relative to the Low CT group, participants in the High CT group had reduced GABA concentrations in the left superior temporal gyrus (STG) voxel. Furthermore, logistic regression showed that participants with low left STG GABA concentrations and low left STG volumes were significantly more likely to be in the high CT group.

**Conclusions:**

This study provides the first evidence that both low GABA concentrations and its interaction with GMV in the left STG are associated with high levels of CT and suggest that altered inhibitory neurotransmission/metabolism may be linked to a lower GMV in the left STG in adults who experienced CT. Future studies are warranted to establish if utilizing these measures can stratify clinical high-risk and predict future clinical outcomes in high CT individuals.

## Introduction

Childhood trauma (CT) is an established antecedent for 29.8% of all disorders worldwide (Kessler et al., 2010), and it is a well-established risk factor for psychiatric disorders, including depression, anxiety, psychosis and psychosis-like experiences and schizophrenia (Aas et al., 2011; Arseneault et al., 2011; Green et al., 2010; Janssen et al., 2004; Paus et al., 2008; Read et al., 2005; Varese et al., 2012; Whitfield et al., 2005). A possible mechanism for this relationship between CT and psychiatric disorders is stress sensitivity, and research in experimental animals has shown that young rodents exposed to adverse events and environments demonstrate a range of aberrant behaviour and physiology in adulthood (e.g. Bonapersona et al., 2019; Smith & Pollak, 2020 for reviews). For example, rodents exposed to abusive maternal behaviours or maternal separation as pups show decreased dendritic arborization, altered synaptic signalling and epigenetic changes throughout the prefrontal cortex, hippocampus and amygdala, as well as anxiety- and depressive-like behaviours in adulthood (Bagot et al., 2009; Danielewicz and Hess, 2014; Lee et al., 2007; Malter Cohen et al., 2013; Monroy et al., 2010; Romeo et al., 2003).

Recently a number of neuroimaging studies in humans have also investigated the effects of CT on adult brain structure. The most robust finding in adult CT populations is lower grey matter volume (GMV) in temporal lobe and prefrontal regions, an effect seen in both psychiatric and non-psychiatric CT populations (e.g. Paquola et al., 2016; Teicher et al., 2018 for reviews). Interestingly, one of the most robust neuroimaging findings in depression, anxiety and psychosis populations, many of whom will have experienced CT (Green et al., 2010; Paus et al., 2008; Read et al., 2005; Varese et al., 2012), is reduced superior temporal gyrus (STG) GMV (Allen et al., 2019; Arnone et al., 2016; Honea et al., 2005; Keshavan et al., 2020; Madonna et al., 2019; Scheepens et al., 2020). Moreover, reduced GMV may be linked to excitotoxicity due to imbalances in local inhibitory and excitatory neurotransmission (Dong et al., 2009; Schobel et al., 2013; Théberge et al., 2007; Zhou & Danbolt, 2014).

In particular, work in experimental animals has shown that early life stressor can lead to a heighted stress response and can affect cortical gamma-aminobutyric acid (GABA)ergic and glutamatergic interneurons and therefore excitatory/inhibitory (E/I) balance (Gomes et al., 2016). For instance, in a neurodevelopmental disruption model, the administration of methylazoxymethanol acetate (MAM) to pregnant rats on gestational day 17 perturbates neurodevelopment and induces histological, anatomical, neurophysiological, pharmacological and cognitive/behavioural alterations on developing paralimbic, frontal and temporal cortices (Gomes et al., 2016; Lodge & Grace, 2011). Of specific importance, MAM rats show a selective loss of parvalbumin-containing interneurons (that contain and release GABA) in both temporal and frontal cortices (Lodge et al., 2009) and the MAM model posits dysregulation of glutamate neurotransmission occurring in the temporal lobe (Lodge & Grace, 2007, 2011; Moore et al., 2006). Additionally, the administration of corticosterone to rats leads to a decrease of mRNA for GAD67 (an enzyme that synthesizes GABA) (Deslauriers et al., 2013; Giovanoli et al., 2013; Stone et al., 2001).

However, there are very few magnetic resonance spectroscopy (^1^H-MRS) studies that have examined brain metabolite concentrations in adult humans that have experienced CT. In the small number of studies that have been conducted, results show that CT is associated with glutamatergic alterations, including lower levels of glutamate, Glx (=glutamate + glutamine), and NAA (N-acetylaspartateglutamate)/Glx ratio in frontal cortex areas (Duncan et al., 2015; Ousdal et al., 2018, 2019; Sonmez et al., 2021) and Glx in the temporal lobe, although only in clinically depressed patients (Poletti et al., 2016). On the other hand, although the association between CT and GABA transmission has not been studied in humans, lower levels of frontal GABA concentrations have been observed in response to other types of adversity in adult populations, including post-traumatic stress disorder (PTSD), trauma exposure and threat-of-shock (Hasler et al., 2010; Sheth et al., 2019).

To date however, no studies have examined the relationship between E/I balance (GABA and glutamate metabolite concentrations) and GMV in an adult CT population. In the current study, because CT is a major risk factor for depression, anxiety and psychosis (Green et al., 2010; Paus et al., 2008; Varese et al., 2012), we chose to examine glutamate and GABA concentrations in the left STG as an altered function, perfusion or structure in this region is one of the most robust neuroimaging findings in these populations (see Allen et al., 2019; Arnone et al., 2016; Madonna et al., 2019; Scheepens et al., 2020), and changes in temporal lobe metabolite concentrations have been reported in these populations (Hjelmervik et al., 2022; Hugdahl et al., 2015; Trzesniak et al., 2008; Venkatraman et al., 2009). In the current study, we aimed to compare left STG GABA and glutamate, as well as whole-brain and left STG GMV in young adults with high and low levels of CT. We predicted that, relative to participants with low CT, a high CT group would show reduced glutamate and GABA concentrations and GMV in the left STG. We further predicted that reduced STG metabolite concentrations and STG GMV would interact to predict high levels of CT. We also conducted an exploratory analysis to examine relationships between STG GMV, metabolite concentrations and clinical measures.

## Method

### Participants

Two hundred and thirty students from Universities of Roehampton and Royal Holloway responded to an online survey via Facebook (delivered on Qualtrics; https://www.qualtrics.com) and were screened using the Childhood Trauma Questionnaire (CTQ) (Bernstein et al., 2003). Fifty-six participants were selected based on the upper and lower quartiles of the sample distribution of the first 100 respondents to establish high CT score (High CT; >40.5, *n* = 29) and low CT score (Low CT; <29.5, *n* = 27) groups. Sensitivity analysis shows that the sample size would allow the detection of a medium effect based on 80% power and an alpha = 0.05.

Exclusion criteria, assessed via a self-report pre-screening survey, included presence of contraindications for magnetic resonance imaging (MRI) scanning (i.e. presence of metal, etc), current use of prescribed medication for neuropsychiatric disorders, or history of or presence of psychiatric and neurological disorders and current use of illicit substances misuses. Absence of psychiatric or neurological diagnosis was assessed with two questions in the screening survey: ‘Have you ever been diagnosed with a psychiatric condition (e.g. Attention Deficit Hyperactivity Disorder [ADHD], depression, anxiety, mood disorders)?’ and ‘Have you ever been diagnosed with a neurological disorder or disease (e.g. epilepsy, stroke, head injury, seizures, brain tumours, brain surgery, Parkinson’s disease)?’ Participants in the Low and High-CT groups were matched for age, gender, estimated IQ, tobacco, cannabis and alcohol use. Participants received £20 for participation. All participants provided informed consent. The research protocol was approved by the Ethical Committee at the University of Roehampton.

### Clinical, IQ and demographic assessment

All participants completed a demographics form (developed in-house) to determine age, sex, level of education, intellectual functioning (assessed via the Wide Range Achievement Test, Reading Level 2; WRAT-R) (Jastak & Wilkinson, 1984), handedness (assessed via the Annett Hand Preference Questionnaire (Annett, 1970), alcohol consumption (units per day), tobacco consumption (cigarettes per day) and cannabis use (assessed via the Cannabis Experience Questionnaire; CEQ) (Barkus & Lewis, 2008). These measures were used to ensure that the High and Low CT groups were matched for these demographic and environmental/lifestyle factors.

The CTQ (Bernstein et al., 2003), a 28-item questionnaire, designed to quantify self-reported CT history in the home, is divided into five clinical subscales: emotional abuse, physical abuse, sexual abuse, emotional neglect and physical neglect. Each item is rated on a 5-point Likert scale (from 1 = never true to 5 = very often true), with higher scores reflecting trauma history. The CTQ demonstrates good test–retest reliability (coefficients ranging from 0.79 to 0.86) and internal consistency (coefficients ranging from 0.66 to 0.92) (Bernstein et al., 2003).

The Depression and Anxiety Stress Scale (DASS) (Lovibond & Lovibond, 1995), a 42-item questionnaire, is designed to quantify the current (state) levels of depression, anxiety, and stress. Each item is rated on a 4-point Likert scale based on symptom severity/frequency. On the depression subscale, a score of 0–9 indicates no depression, 10–13 mild depression, 14–20 moderate depression, 21–27 severe depression, and 28 + extremely severe depression. On the anxiety subscale, a score of 0–7 indicates no anxiety, 8–9 mild anxiety, 10–14 moderate anxiety, 15–19 severe anxiety, and 20 + extremely severe anxiety. On the stress subscale, a score of 0–14 indicates no stress, 15–18 mild stress, 19–25 moderate stress, 26–33 severe stress, and 34 + extremely severe stress.

We also used the Connor–Davidson Resilience Scale (CD-RISC-25) (Connor & Davidson, 2003), a 25-item questionnaire, to measure resilience. Each item is rated on a 5-point scale (0–4), with higher scores reflecting greater resilience.

### MRI acquisition

All MRI scans were acquired on a 3T Siemens Magnetom TIM Trio scanner using a 32-channel head coil at the Combined Universities Brain Imaging Centre (http://www.cubic.rhul.ac.uk/). Structural T1-weighted magnetization-prepared rapid acquisition gradient echo images were acquired with a spatial resolution of 1 mm × 1 mm × 1 mm, in a plane resolution of 256 × 256 × 176 continuous slices and scanning time of approximately 5 min.

### ^1^H-MRS data acquisition and analysis

**Figure 1.**
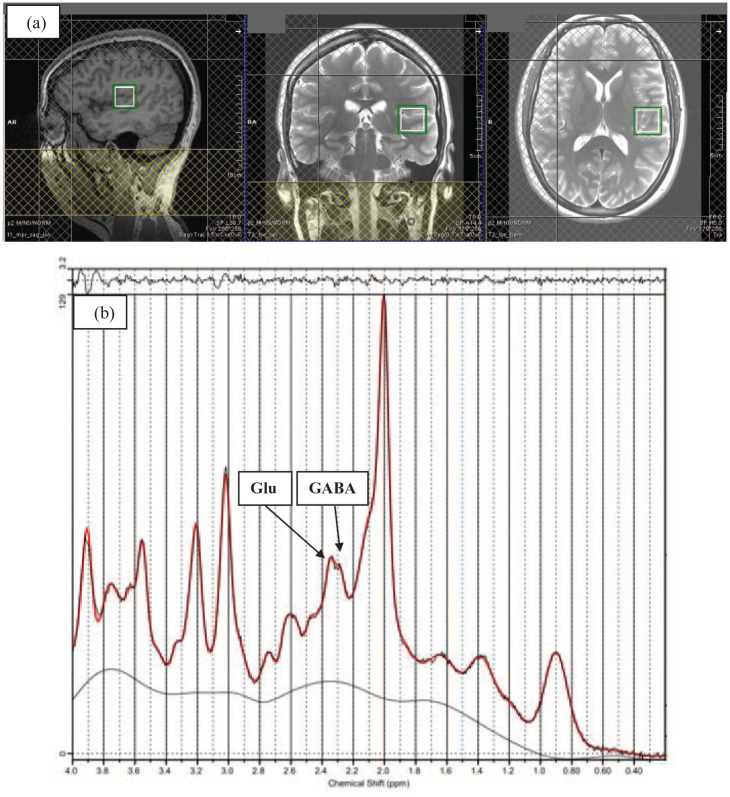
(a) Example of ^1^H-MRS voxel placement in the left superior temporal gyrus (STG) (sagittal, coronal, and axial orientations) (b) ^1^H-MRS spectrum obtained from the voxel in A (black line) and the overlay of the spectral fit (red line) (see the Supplemental Figure S1 for another example).

^1^H-MRS in vivo spectra were acquired from a 20 × 20 × 20 mm voxel located in the left STG during rest (see Figure 1). The voxel was positioned manually in the left STG by reference to an axial T1-weighted gradient echo image. Spectra were acquired using SPin ECho full Intensity-Acquired Localized spectroscopy (SPECIAL) (Mlynárik et al., 2006). The ^1^H-MRS sequence was acquired with water suppression (TR 3000 ms, TE 8.5 ms, Phase cycle Auto, 192 averages from the STG voxel) in each participant (Godlewska et al., 2015). Water unsuppressed spectra (16 averages) were also acquired. Six outer volume suppression slabs were applied (one on each side at 5 mm from the edge of the cubic voxel) to suppress signals originating from outside the volume of interest and to minimize motion-related image-selected in vivo spectroscopy subtraction artefacts. Spectra were analysed using LCModel 6.3-1L with the basis set consisting of 19 simulated basis spectra: alanine (Ala), ascorbate (Asc), aspartate (Asp), creatine (Cr), GABA, glucose (Glc), glutamine (Gln), glutamate (Glu), glycine (Gly), glutathione (GSH), glycerophosphocholine (GPC), phosphocholine (PCh), lactate (Lac), myo-inositol (mI), N-acetylaspartate (NAA), N-acetylaspartateglutamate (NAAG), phosphorylethanolamine (PE), scyllo-inositol (Scyllo) and taurine (Tau).

The basis set was simulated using FID-A (Simpson et al., 2017), for TE = 8.5 ms, magnetic field strength = 3T and assuming ideal RF pulses. We excluded spectra with Cramér- Rao lower bounds (CRLB) > 20% as reported by LCModel. In addition to metabolite levels, line widths and signal-to-noise ratios were estimated by LCModel. All spectra had a line width <8 Hz (estimates of the line widths produced by the LC model software) and a mean signal-to-noise ratio (SNR) > 39.72 which are within the accepted ranges (Godlewska et al., 2015; Hollestein et al., 2021). SNR is defined as is defined the ratio of the maximum in the spectrum-minus baseline over the analysis window to twice the root-mean-square (rms) residuals. Following these quality control checks, we reported results form 51 (25 Low CT and 26 High CT) and 56 (27 Low CT and 29 High CT) participants for STG GABA and STG Glu, respectively.

Water referencing and eddy current correction were used to quantify metabolite levels. When quantified in this way, metabolite levels are influenced by cerebral spinal fluid (CSF), grey (GM) and white (WM) matter volumes of the region in which spectra are obtained within the voxel (Srinivasan et al., 2006) and inter-individual differences in cortical grey matter (Huster et al., 2007). In order to account for these confounds, we used the T1-weighted anatomical images to estimate the GM and WM content of the STG voxel in which the ^1^H-MRS measures were performed using GABA Analysis Toolkit (Gannet 2.0, https://github.com/markmikkelsen/Gannet) adapted to work with Siemens SPECIAL data. The segmentation was performed using ‘new segment’ in SPM 8 (http://www.fil.ion.ucl.ac.uk/spm/software/spm8/). CSF, GM volume and WM volume were then accounted for in the expression of GABA and Glu levels using LCModel (Ernst et al., 1993); corrected metabolite levels will be referred to as *Glu Corr* and *GABA Corr* using the formula *Glu Corr* = (Glu*(43300*GMV + 35880*WMV + 55556*CSF))/(35880* (1-CSF)) and GABA Corr = (GABA*(43300*GMV + 35880*WMV + 55556*CSF))/(35880*(1-CSF)). Relaxation corrections were not applied apart from correcting for tissue water relaxation, assuming T_2_ = 80 ms, by using LCModel parameter ATTH2O = 0.899.

IBM^®^ SPSS Statistics Version 26 and Jamovi 2.2.5 were used for data analysis. Low CT and High CT groups were compared on demographic and clinical measures, as well as STG metabolite levels, SNR, line width and CRLB by using chi-square or independent sample *t*-tests. Logistic regression analyses were also conducted to predict the CT group (high vs low) from SFG GABA *Corr* and Glu *Corr* levels. Relationships between clinical measures and GABA *Corr* and Glu *Corr* metabolite concentrations were analysed using bivariate correlations. A statistical significance threshold of *p* < 0.05 was applied throughout.

### VBM and region-of-interest analysis

Images were analysed using Computational Anatomy Toolbox 12 (CAT12; http://www.neuro.uni-jena.de/cat) implemented in SPM12 (Wellcome Trust Centre for Neuroimaging; www.fil.ion.ac.uk/spm/software/spm12). As per standard protocol (see http://www.neuro.uni-jena.de/cat12/CAT12-Manual.pdf), data were skull-stripped using the adaptive probability region-growing approach, normalized to the standard tissue probability map, and segmented into grey matter, white matter, and CSF. These images were ‘modulated normalized’ images (i.e. voxel values were modulated using the Jacobian determinant), derived from the spatial normalization so that the absolute volume of grey matter could be compared between groups. This type of modulation requires group analyses to correct for individual differences in brain size; total intracranial volume was therefore added as a covariate to all group-level general linear models. The images were then registered to the MNI template using DARTEL registration and smoothed using an 8-mm Gaussian Kernel. Data quality was checked based on the image quality ratings (IQR) generated by CAT12, which factors in both noise (e.g. motion) and spatial resolution. The visual inspection revealed no issues. Only the images where the IQR was above the ‘good’ threshold (i.e. B-; 0.80) were included in the analyses; hence, the reported results for VBM and region-of-interest (ROI) analyses are from 49 participants (22 Low CT and 27 High CT).

In order to examine whole-brain level GM volume differences between High CT and Low CT groups, two-sample *t*-tests that control for TIV were used to determine brain regions in which GMV differed between Low CT group and High CT group. A threshold of *p* < 0.05 with family-wise error correction for multiple comparisons was applied to all contrasts.

As CAT12 also enables the estimation of mean tissue volumes for different volume-based atlas maps, we used a ROI labelling approach that parcellates each brain into several anatomical regions according to the neuromorphometric atlas (provided by Neuromorphometrics, Inc. (http://Neuromorphometrics.com)) to estimate the sum of local GM inside the left STG. IBM^®^ SPSS Statistics Version 26 was used for data analysis. Low CT and High CT groups were compared on the left IFG GM volume by an independent sample *t*-test. Relationships between clinical measures, the left STG GABA *Corr* and Glu *Corr* metabolite concentrations, and the left STG GM volume were analysed using partial correlations adjusted for TIV. Logistical regression models were also used to test whether the interaction between the STG GMV and metabolite concentrations predicted CT group membership. A statistical significance threshold of *p* < 0.05 was applied throughout.

## Results

### Participant characteristics

Due to the slightly differing group configurations for GABA *Corr* and Glu *Corr* concentrations and GMV resulting from quality control checks, results are reported separately. Table 1 provides a full summary of participant characteristics in Low and High CT groups for the analysis of GABA *Corr* and Glu *Corr* metabolite concentrations and GMV in the left STG. The Low and High CT groups were matched for sex, age, WRAT-R estimated IQ, years in education, tobacco use, alcohol use, cannabis use and handedness, but by design differed significantly on measures of CT. As expected, the Low and High CT groups also differed significantly on measures of depression, anxiety and stress. There was also a difference for CD-RISC (resilience) scores for the participants in the Glu *Corr* and GMV analyses. Groups did not differ on GM, WM and CSF tissue volumes in the STG voxel.

**Table 1.** Demographic summary, questionnaire measures and tissue maps in the Low CT and High CT groups for GABA Corr, Glu Corr metabolite and GMV analysis.

Characteristic	GABA Corr				Glu Corr				STG GMV			
	Low CT (*n* = 25)	High CT (*n* = 26)	*t/χ* ^2^	*p*	Low CT (*n* = 27)	High CT (*n* = 29)	*t/χ* ^2^	*p*	Low CT (*n* = 22)	High CT (*n* = 27)	*t/χ* ^2^	*p*
Sex (M/F)	8/17	6/20	0.51	0.475	8/19	6/23	0.59	0.440	6/16	6/21	0.17	0.683
Age	19.92 (1.66)	20.69 (1.49)	−1.75	0.086	20.00 (1.61)	20.79 (1.80)	−1.73	0.089	20.14 (1.70)	20.67 (1.52)	−1.15	0.127
WRAT-R IQ	75.72 (5.70)	75.42 (4.47)	0.21	0.837	75.92 (5.56)	75.21 (4.57)	0.53	0.598	75.55 (5.96)	75.89 (3.93)	−0.24	0.405
Education, years	14.88 (3.27)	15.96 (1.84)	−1.46	0.150	15.03 (3.20)	15.38 (3.28)	−0.39	0.695	15.59 (2.02)	15.96 (1.79)	−0.69	0.248
CTQ-total	26.88 (1.42)	57.54 (12.51)	−12.18	<0.001	26.77 (1.42)	57.21 (12.22)	−12.85	<0.001	26.73 (1.49)	56.93 (12.58)	−11.17	<0.001
Emotional abuse	5.76 (0.97)	14.38 (3.89)	−10.77	<0.001	5.70 (0.95)	14.31 (3.82)	−11.38	<0.001	5.55 (0.86)	14.19 (3.89)	−10.19	<0.001
Physical abuse	5.00 (0.00)	9.96 (5.02)	−4.94	<0.001	5.00 (0.00)	9.76 (4.78)	−5.17	<0.001	5.00 (0.00)	10.00 (4.85)	−4.82	<0.001
Sexual abuse	5.00 (0.00)	7.65 (4.63)	−2.86	0.006	5.00 (0.00)	7.66 (4.49)	−3.07	0.003	5.00 (0.00)	7.48 (4.48)	−2.60	0.006
Emotional neglect	5.96 (1.40)	14.77 (3.84)	−10.80	<0.001	5.92 (1.35)	14.79 (3.83)	−11.38	<0.001	6.00 (1.45)	14.74 (3.77)	−10.26	<0.001
Physical neglect	5.16 (0.37)	10.77 (3.65)	−7.65	<0.001	5.14 (0.36)	10.69 (3.55)	−8.07	<0.001	5.18 (0.39)	10.52 (3.62)	−6.87	<0.001
CD-RISC	69.60 (8.47)	62.19 (17.28)	1.93	0.059	69.59 (8.54)	61.69 (18.04)	2.07	0.043	70.59 (7.83)	62.07 (17.86)	2.08	0.022
DASS_Depression	3.04 (3.56)	11.23 (7.52)	−4.94	<0.001	3.56 (3.91)	11.90 (9.29)	−4.32	<0.001	3.23 (3.48)	11.67 (9.19)	−4.07	<0.001
DASS_Anxiety	7.76 (5.77)	13.50 (7.78)	−2.98	0.004	3.30 (3.22)	9.38 (7.73)	−3.79	<0.001	2.91 (3.10)	8.81 (7.20)	−3.58	<0.001
DASS_Stress	3.16 (3.30)	9.04 (7.98)	−3.41	<0.001	7.44 (5.56)	14.07 (7.90)	−3.58	<0.001	6.59 (5.60)	13.56 (7.64)	−3.56	<0.001
Tobacco use*	1.55 (3.85)	0.08 (0.28)	1.87	0.069	1.40 (3.68)	0.37 (1.48)	1.32	0.195	0.81 (2.12)	0.37 (1.48)	0.812	0.211
Alcohol use**	1.73 (2.34)	1.44 (1.90)	0.48	0.634	1.78 (2.29)	1.50 (1.94)	0.50	0.617	1.96 (2.41)	1.57 (1.98)	0.621	0.269
CEQ	3.20 (7.08)	2.19 (3.45)	0.65	0.519	3.03 (6.83)	2.17 (3.34)	0.61	0.545	3.18 (7.22)	2.11 (3.41)	0.684	0.498
Handedness (R/L)	20/5	23/3	0.69	0.406	21/6	26/3	1.46	0.227	6/16	24/3	2.11	0.146
GM volume	0.63 (0.08)	0.62 (0.08)	0.50	0.620	0.63 (0.08)	0.62 (0.08)	0.44	0.665	0.63 (0.08)	0.62 (0.08)	0.241	0.405
WM volume	0.20 (0.14)	0.25 (0.14)	−1.21	0.233	0.21 (0.14)	0.25 (0.14)	−1.06	0.293	0.23 (0.14)	0.25 (0.14)	−0.544	0.294
CSF volume	0.17 (0.11)	0.13 (0.09)	1.27	0.211	0.16 (0.11)	0.13 (0.09)	1.16	0.253	0.14 (0.10)	0.13 (0.09)	0.576	0.284

CD-RISC: The Connor–Davidson Resilience Scale; CEQ: Cannabis Experience Questionnaire; CSF: cerebro-spinal fluid; CTQ: Childhood Trauma Questionnaire; DASS: Depression, Anxiety, Stress Scale; F: female; GABA: gamma-aminobutyric acid; GM: grey matter; GMV: grey matter volumes; L: left; M: male; R: right; S: state; STAI: State Trait Anxiety Inventory; STG: superior temporal gyrus; T: trait; WM: white matter; WRAT-R: Wide Range Achievement Test-Revised.

* cigarettes/d. ** units/d.

### GABA Corr and Glu Corr metabolite concentrations

**Figure 2.**
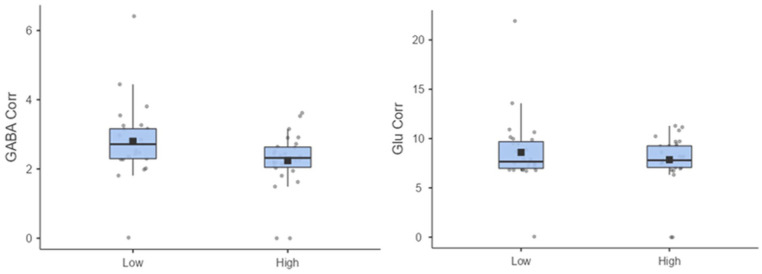
(Left) Superior temporal gyrus (STG) gamma-aminobutyric acid (GABA) Corr (Right) STG Glu Corr levels by CTQ groups in institutional units. All datapoints are within the expected quality control parameters.

STG GABA *Corr* and Glu *Corr* metabolite levels and spectra quality control data for Low and High CT groups are reported in Table 2. All other metabolite levels (NAA, Cr, mI, Glx) are reported in Supplemental Table S1. No significant differences between groups were detected for SNR, line width, or CRLB. The difference between Low and High CT groups for STG Glu *Corr* was non-significant (*p* > 0.1). However, the High CT group (*M* = 2.24, SD = 0.83 institutional units) had significantly lower STG GABA *Corr metabolite* concentrations compared to the Low CT group (*M* = 2.79, SD = 1.10 institutional units), (*t(49*) = 2.03. *p* = 0.048; Figure 2). After controlling for depression, anxiety, stress and resilience, logistic regression analysis revealed that STG GABA *Corr* was a significant fit to the model, *χ2(5)* = 28.66, *p* *<* 0.001, Cox and Snell’s *R*^2^ = 0.43, Nagelkerke’s *R*^2^ = 0.57 and a significant predictor of CTQ group membership (*b* = −1.08, SE = 0.05, *z* = 3.08, *p* = 0.048, CI = [0.12, 0.99]). However, STG Glu *Corr* was not a significant predictor of CTQ group membership (*p* > 0.05).

**Table 2.** Means, standard deviations and statistical analyses for ^1^H-MRS quality control measures and STG GABA Corr and Glu Corr levels by Low and High CTQ groups.

	Low CT	High CT	*t*	*p*
GABA *Corr* (IU)	2.79 (1.10)	2.24 (0.83)	2.03	0.048
SNR	44.60 (11.64)	42.95 (6.86)	0.59	0.559
Line width (Hz)	5.02 (1.85)	4.62 (1.16)	0.90	0.372
GABA CRLB (%)	13.52 (2.72)	15.07 (2.92)	−1.96	0.055
Glu *Corr* (IU)	8.60 (3.52)	7.85 (2.56)	0.90	0.368
SNR	44.73 (11.54)	39.72 (11.30)	1.63	0.110
Line width (Hz)	5.06 (1.83)	5.72 (3.16)	−0.94	0.350
Glu CRLB (%)	5.25 (2.65)	5.06 (1.16)	0.35	0.726

Corr: corrected; CRLB: Cramér-Rao lower bounds; CT: childhood trauma; GABA: gamma-aminobutyric acid; GLU: Glutamate; 1H-MRS: underwent magnetic resonance spectroscopy; Hz: hertz; IU: institutional units; SNR: signal-to-noise ratio; STG: superior temporal gyrus.

### Associations between GABA Corr and Glu Corr metabolite concentrations and clinical measures

Correlations between metabolite concentrations and clinical measures in the High CT group were non-significant (all *ps* > 0.05). Results for both groups are reported in Supplemental Table S2.

### VBM analysis

Following whole-brain VBM analysis, for both contrasts (Low CT > High CT and High CT > Low CT), no GM volume differences were found. The left STG ROI analysis showed that the difference between Low and High CT groups for left STG GM volume was also non-significant (*t(54)* *=* 0.392, *p* = 0.697).

### Associations between the left STG GM volume and GABA Corr and Glu Corr concentrations

After controlling for TIV, partial correlations revealed positive associations between the left STG GM volume and (1) GABA *Corr* (*r*(17) = 0.502, *p* = 0.029), and (2) Glu *Corr* (*r*(22) = 0.478, *p* = 0.028) metabolite concentrations in the low CT group, but not in the high CT group (all *ps* > 0.05).

**Figure 3.**
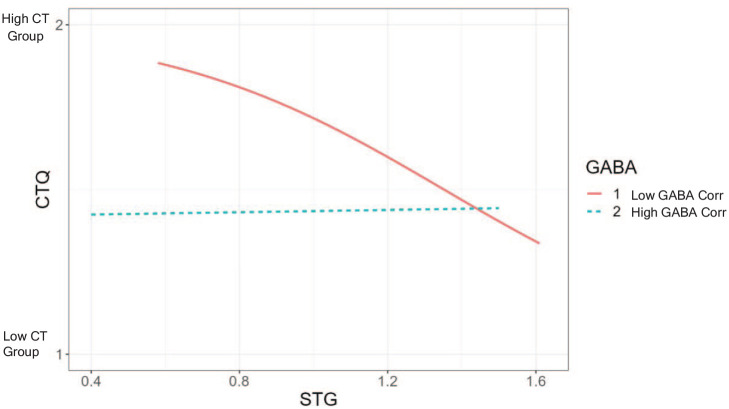
Logistic regression showing an interaction between the left superior temporal gyrus (STG) GM volume and gamma-aminobutyric acid (GABA) Corr levels.

TIV-controlled logistic regression showed that the interaction between GABA *Corr* levels (categorized as low and high GABA *Corr* by using a median split) and the left STG GM volume was a significant fit of the model, *χ*^2^(2) = 7.16, *p* < 0.03, Cox and Snell’s *R*^2^ = 0.15, Nagelkerke’s *R*^2^ = 0.20 and a significant predictor of CTQ group membership (*b* = −0.95, SE = 0.43, z = 4.69, *p* < 0.03). Figure 3 shows that participants with low left STG GABA *Corr* levels and low left STG volumes were significantly more likely to be in the high CTQ group (95%CI 0.17–0.92). The same model using Glu *Corr* revealed a non-significant result (*p* > 0.05).

### Associations between the left STG GM volume and clinical measures

By using the estimates of the sum of local grey matter inside the left STG ROI, partial correlations (TIV-controlled) revealed a positive association between the CD-RISC scores and the left STG GM volume in the low CT group (*r*(22) = 0.484, *p* = 0.026), but not in the high CT group (*p* > 0.1). In the low CT group, the left STG GM volume was also found to be negatively associated with DASS-Depression scores (*r*(19) = −0.451, *p* = 0.040). All other associations between the left STG volume and clinical measures were non-significant (all *ps* > 0.05). Results for both groups are reported in Supplemental Table S3.

## Discussion

To our knowledge, this is the first study that has investigated the left STG GABA and glutamate, as well as the whole brain and the left STG GM volume differences in young adults in high and low levels of CT. Partially in line with our prediction, we found that individuals in the high CT group had reduced levels of the left STG GABA, but not glutamate, compared to individuals in the low CT group. Furthermore, having lower levels of GABA predicted high CT group membership. Whilst we did not observe any differences between High and Low CT groups in terms of left STG GMV, reduced levels of left STG GABA *and* lower left STG GMV interacted to predict high CT group membership. Our exploratory analysis also showed that the left STG GMV was positively associated with GABA and glutamate levels, resilience (CD-RISC scores) and negatively associated with depression (DASS-Depression scores) in the low CTgroup, but not in the high CT group.

Our observation of reduced GABA (but not glutamate) levels in the high (vs low) CT group is consistent with experimental work in animals showing the impact of early life adversity on GABA concentrations (Gomes et al., 2016). As no previous studies have linked measures of CT and GABAergic function in healthy humans, we also extended the current clinical evidence that has shown associations between CT and frontal glutamatergic alterations (Duncan et al., 2015; Ousdal et al., 2018, 2019; Sonmez et al., 2021). Our findings of reduced STG GABA metabolite concentration in adults that have experienced CT are also broadly in line with a study in patients with PTSD and trauma exposure (without PTSD) that showed reduced frontal (Sheth et al., 2019) and temporal GABA concentrations (Meyerhoff et al., 2014). Additionally, not only chronic but also acute forms of stress have been shown to decrease prefrontal GABA concentrations by 18% in humans (Hasler et al., 2010). Together, these findings may reflect a selective loss of parvalbumin-containing interneurons (that contain and release GABA) (Lodge et al., 2009) and/or a decrease of mRNA for GAD67 (an enzyme that synthesizes GABA) (Deslauriers et al., 2013; Giovanoli et al., 2013; Stone et al., 2001) in the left STG of young adults that have experienced CT. It might also be speculated that the reduction of the left STG GABA in adults that have experienced CT would reduce GABA’s inhibitory influence on neural circuits involved in responding to stress and/or threat. Converging evidence showing hyperactivity in the limbic structures in individuals who were exposed to early stress and/or childhood maltreatment (Teicher et al., 2003) supports this view.

In terms of our null glutamate finding, it may be the case that CT may selectively alter frontal (but not temporal) glutamatergic function (as evidenced in Duncan et al., 2015; Ousdal et al., 2018, 2019; Sonmez et al., 2021) and temporal GABAergic (as evidenced in the current study) mechanisms separately in healthy individuals. In fact, based on animal studies, it has been shown that stress selectively attenuates (1) excitatory tone (i.e. glutamatergic activity) in the frontal area but not in the amygdala or in the temporal lobe (Knox et al., 2010) and (2) inhibitory tone (i.e. GABAergic activity) in the temporal areas (De Groote & Linthorst, 2007). Moreover, these findings suggest that cortical glutamate levels might be perturbed in high (vs low) CT groups only under specific circumstances. For instance, Duncan et al. (2015) showed that the CTQ scores and frontal glutamate levels correlated with neural BOLD responses to the anticipation of aversive stimuli in areas covering the prefrontal-insular-motor cortex network, suggesting that left STG glutamate might be selectively altered only in the context of affective functioning.

It is important to note that, MEshcher-GArwood Point-RESolved Spectrosocpy (MEGA-PRESS) (Mescher et al., 1998) is the most widely used MRS acquisition protocol, with reproducible within- and between-session GABA measurement at 3T (Baeshen et al., 2020; Brix et al., 2017). However, in the current study and previous studies (Faulkner et al., 2021; Kozhuharova et al., 2021; Godlewska et al., 2015; Morgenroth et al., 2019), SPECIAL was utilized with promising results. Additionally, it has been shown that both GABA (Near et al., 2013) and other metabolite levels (e.g. GSH) (Wijtenburg et al., 2019) were comparable between SPECIAL and more conventional spectral editing techniques – albeit by using larger voxels; hence, we encourage researchers to replicate our findings by using other sequences.

Unlike previous studies, although we did not observe temporal GMV differences between High and Low CT groups (Paquola et al., 2016; Teicher et al., 2018), we found that the left STG GMV was (1) positively associated with GABA and glutamate concentrations as well as resilience, and (2) negatively associated with depression only in the low CT group. Furthermore, we found out that, lower left STG GMV and lower levels of GABA metabolite concentrations interacted to predict high CT group membership. Whilst this finding is difficult to interpret, it is possible that lower left STG GMV and GABA levels are linked to decreased resilience and increased levels of negative affect, such as depression. This may play a role in the risk or the development of psychiatric (or psychiatric-like) symptoms following early traumatic experiences and vulnerability to psychiatric conditions in later life (Nelson et al., 2020).

There are several notable limitations in the current research. First of all, based on the generic power analyses, although the sample size was adequate to detect medium-to-large effects, our results would benefit from replication in a larger sample, which could be achieved by combining ^1^H-MRS and morphometric data from multiple centres. Secondly, as the CTQ relies upon autobiographical recall that may be biased by current affective states (Vrijsen et al., 2017), and our definition of High and Low CT groups was also arbitrary (i.e. based on the upper and lower quartiles of the 100 first respondents), though not unusual (e.g. Kim et al., 2018), our results must be interpreted with caution. Thirdly, classification of participants based on the total CTQ score (as in the current study) may hinder the potential impact that trauma subtype (sexual abuse, physical abuse, emotional abuse, sexual neglect and physical neglect), severity and duration (single vs prolonged trauma) may have on brain volume and chemistry. Fourthly, given the dimensions and orientation of our ^1^H-MRS voxel, other temporal lobe structures might have also been included in the voxel, which may have confounded our results. Fifthly, although the CRLB GABA values in the current study are consistent with previous studies (Kozhuharova et al., 2021; Weis et al., 2021) and are within standard limits (Oz et al., 2020), as GABA is a small signal and given the tendency for the high CT group to have higher CRLB for GABA, the possibility of differences in spectral quality driving the results should not be underestimated. Sixthly, by using conventional ^1^H-MRS, one cannot simply determine whether differences in neurometabolite levels are associated with neurotransmission or metabolism; hence, future research should utilise more sophisticated MRS protocols (Jelen et al., 2018) to address this issue. Seventhly, as some participants were excluded from the volumetric analysis based on image quality, we cannot exclude the possibility that image quality might have affected the tissue segmentation within the MRS voxel. Finally, due to the cross-sectional nature of our study, we could not determine cause and effect relationships; therefore, further longitudinal studies are warranted in order to allow stronger causal inferences to examine the effects of CT on brain volume and chemistry.

In conclusion, our findings suggest that early traumatic experiences are associated with lower left STG GABA neurotransmission/metabolism. Furthermore, although we did not observe a direct group effect in left STG GMV, traumatic stress may influence the left STG GMV through excitotoxicity due to GABA alterations. Further longitudinal studies are warranted to identify neurochemical and neurostructural (and their interaction) correlates of CT throughout development and in populations with other psychiatric disorders, with an aim towards contributing to pharmacological treatments of stress-related mental illness.

## Supplemental Material

sj-docx-1-jop-10.1177_02698811231168243 – Supplemental material for Signatures of exposure to childhood trauma in young adults in the structure and neurochemistry of the superior temporal gyrus Click here for additional data file. Supplemental material, sj-docx-1-jop-10.1177_02698811231168243 for Signatures of exposure to childhood trauma in young adults in the structure and neurochemistry of the superior temporal gyrus by Piril Hepsomali, Sandra Machon, Holly Barker, David J Lythgoe, Kenneth Hugdahl, Maria Gudbrandsen and Paul Allen in Journal of Psychopharmacology
