# Supplementary material for: Signatures of exposure to childhood trauma in young adults in the structure and neurochemistry of the superior temporal gyrus
Source: J Psychopharmacol. 2023 Apr 17;37(5):510–9. doi: 10.1177/02698811231168243 (PMC10185913; doi:10.1177/02698811231168243)
Supplement: sj-docx-1-jop-10.1177_02698811231168243 – Supplemental material for Signatures of exposure to childhood trauma in young adults in the structure and neurochemistry of the superior temporal gyrus [file sj-docx-1-jop-10.1177_02698811231168243.docx]

**Table s1.** Means, standard deviations and statistical analyses for 1H-MRS quality control measures, left STG NAA, Cr, mI, and Glx levels by low and high CT groups

|  | **Low CT** | **High CT** | ***t*** | ***p*** |
| --- | --- | --- | --- | --- |
| NAA (IU) | n= 28 | n= 28 |  |  |
|  | 6.66 (1.15) | 6.37 (0.73) | 1.110 | .271 |
| Cr (IU) | n= 26 | n= 29 |  |  |
|  | 7.30 (1.87) | 7.33 (6.34) | -.018 | .986 |
| mI (IU) | n= 26 | n= 27 |  |  |
|  | 4.40 (0.61) | 4.56 (0.62) | -.981 | .331 |
| Glx (IU) | n= 27 | n= 29 |  |  |
|  | 7.27 (2.01) | 7.51 (1.12) | -.566 | .574 |

*NAA: N-acetylaspartate; IU: institutional units; Cr: Creatine; mI:* *myo-inositol, Glx:a combined measure of glutamate and glutamine*

**Table s2.** Pearson Correlation Coefficients of the associations between left STG GABA Corr and Glu Corr levels and clinical measures by low and high CT groups

|  | **Left STG GABA *Corr*** | |
| --- | --- | --- |
|  | **Low CT** | **High CT** |
| CTQ | -0.090 | 0.370 |
| CD-RISC | 0.087 | 0.145 |
| DASS_Depression | 0.081 | -0.001 |
| DASS_Stress | -0.226 | 0.073 |
| DASS_Anxiety | 0.035 | 0.132 |
|  | **Left STG Glu *Corr*** | |
|  | **Low CT** | **High CT** |
| CTQ | 0.023 | 0.222 |
| CD-RISC | -0.054 | -0.016 |
| DASS_Depression | 0.050 | 0.180 |
| DASS_Stress | -0.201 | 0.255 |
| DASS_Anxiety | 0.013 | 0.181 |

**Correlation is significant at the 0.01 level (2-tailed)

*Correlation is significant at the 0.05 level (2-tailed)

**Table s3.** Pearson Correlation Coefficients of the associations between left STG GM volume and clinical measures by low and high CT groups

|  | **Left STG GM volume** | |
| --- | --- | --- |
|  | **Low CT** | **High CT** |
| CTQ | -0.053 | 0.063 |
| CD-RISC | 0.484* | 0.209 |
| DASS_Depression | -0.451* | -0.183 |
| DASS_Stress | -0.304 | 0.209 |
| DASS_Anxiety | -0.286 | 0.062 |

**Correlation is significant at the 0.01 level (2-tailed)

*Correlation is significant at the 0.05 level (2-tailed)

**Figure s1.**^1^H-MRS spectrum obtained from the left STG voxel (black line) and the overlay of the spectral fit (red line)
